# Artificial axons as a biomimetic 3D myelination platform for the discovery and validation of promyelinating compounds

**DOI:** 10.1038/s41598-023-44675-6

**Published:** 2023-11-09

**Authors:** Anna Jagielska, Kristin Radzwill, Daniela Espinosa-Hoyos, Mingyu Yang, Kavin Kowsari, Jonathan E. Farley, Stefanie Giera, Ann Byrne, Guoqing Sheng, Nicholas X. Fang, James C. Dodge, Carlos E. Pedraza, Krystyn J. Van Vliet

**Affiliations:** 1 Department of Materials Science and Engineering, Massachusetts Institute of Technology, Cambridge, MA USA; 2 Sanofi, Cambridge, MA USA; 3 Department of Chemical Engineering, Massachusetts Institute of Technology, Cambridge, MA USA; 4 Harvard-MIT Health Sciences and Technology, Massachusetts Institute of Technology, Cambridge, MA USA; 5 Department of Mechanical Engineering, Massachusetts Institute of Technology, Cambridge, MA USA; 6 Department of Biological Engineering, Massachusetts Institute of Technology, Cambridge, MA USA; 7 Present Address: Artificial Axon Labs, Boston, MA USA; 8 Present Address: Sanofi, Cambridge, MA USA; 9 Present Address: Alnylam Pharmaceuticals, Cambridge, MA USA; 10 Present Address: Merck, Rahway, NJ USA; 11 Present Address: AstraZeneca, Boston, USA; 12 Present Address: The University of Hong Kong, Hong Kong, China; 13 Present Address: Sai, Boston, MA USA; 14 Present Address: Cornell University, Ithaca, NY USA

**Keywords:** Regeneration and repair in the nervous system, Oligodendrocyte, Multiple sclerosis, Phenotypic screening, Multiple sclerosis

## Abstract

Multiple sclerosis (MS), a chronic neurodegenerative disease driven by damage to the protective myelin sheath, is currently incurable. Today, all clinically available treatments modulate the immune-mediated symptoms of the disease but they fail to stop neurodegeneration in many patients. Remyelination, the regenerative process of myelin repair by oligodendrocytes, which is considered a necessary step to protect demyelinated axons and stop neuronal death, is impaired in MS patients. One of the major obstacles to finding effective remyelinating drugs is the lack of biomimetic drug screening platforms that enable quantification of compounds’ potential to stimulate 3D myelination in the physiologically relevant axon-like environment. To address this need, we built a unique myelination drug discovery platform, by expanding our previously developed technology, artificial axons (AAs), which enables 3D-printing of synthetic axon mimics with the geometry and mechanical properties closely resembling those of biological axons. This platform allows for high-throughput phenotypic myelination assay based on quantification of 3D wrapping of myelin membrane around axons in response to compounds. Here, we demonstrate quantification of 3D myelin wrapping by rat oligodendrocytes around the axon mimics in response to a small library of known pro-myelinating compounds. This assay shows pro-myelinating activity for all tested compounds consistent with the published in vitro and in vivo data, demonstrating predictive power of AA platform. We find that stimulation of myelin wrapping by these compounds is dose-dependent, providing a facile means to quantify the compounds’ potency and efficacy in promoting myelin wrapping. Further, the ranking of relative efficacy among these compounds differs in this 3D axon-like environment as compared to a traditional oligodendrocyte 2D differentiation assay quantifying area of deposited myelin membrane. Together, we demonstrate that the artificial axons platform and associated phenotypic myelin wrapping assay afford direct evaluation of myelin wrapping by oligodendrocytes in response to soluble compounds in an axon-like environment, providing a predictive tool for the discovery of remyelinating therapies.

## Introduction

Myelination is a critical developmental process in which glial cells such as oligodendrocytes wrap myelin around neuronal axons. Myelin is a lipid-rich material that forms an insulative sheath around axons, thus facilitating accelerated signal propagation along the axon’s length. Both the improper formation of myelin during development and the destruction of healthy myelin can lead to debilitating diseases with wide-ranging sensory and motor symptoms^1–4^. In demyelinating diseases such as multiple sclerosis (MS), the loss of myelin can result in impaired neuronal signal transmission, axon loss, and neurodegeneration.

There is currently no cure available for MS. Most available treatments for MS are disease-modifying drugs that reduce or halt the autoimmune symptoms of the disease, but fail to reverse the potential long-term degeneration caused by myelin loss^5,6^. One promising area of exploration is the discovery of drugs that promote remyelination, which is the regenerative process of myelin repair by endogenous oligodendrocytes and oligodendrocyte progenitor cells (OPCs)^7^.

A major obstacle to discovering remyelinating drugs is the lack of tools that enable visualization and quantification of myelin wrapping in vitro, in the environments that provide sufficient similarity to the conditions in the brain. Current methods to visualize and quantify myelin in vitro include co-culturing oligodendrocytes and neurons on tissue-culture polystyrene (TCPS) dishes^8^. Although such methods can recapitulate 3D myelin wrapping, they involve lengthy and complex experimental flow, have inherently low reproducibility, and therefore have limited application for drug screening. Other approaches include fabricating cell-free physical models of axons from glass or glassy polymers, formats that have an effective stiffness several orders of magnitude higher than biological axons^9–14^, which are characterized by very low, kilopascal-scale stiffness (Young’s modulus)^15^. For example, Mei et al. developed arrays of printed glass cones used to screen a library of small molecules for their ability to stimulate myelin wrapping^9^. Although this system enabled high throughput compounds screening, it provided the environment with gigapascal mechanical stiffness of glass, and the conical geometries significantly different from the cylindrical, high aspect ratio geometry of biological axons. Similarly, commercially available or custom made electrospun fibers^10–14^ made of polystyrene, polycaprolactone (PCL) or poly-L-lactic acid (PLLA) provide mega-to-gigapascal unphysiological mechanical stiffness. We and others have shown that cells of the central nervous system (CNS), including oligodendrocytes, are sensitive to the biophysical and geometric cues in their environment^16–20^, which can affect various aspect of these cell biology, including proliferation, migration, differentiation and myelination^16–19^. Therefore, the response of cells to applied cues in these unphysiological in vitro conditions may not be representative of the responses expected in the CNS. In our recent, related study (preprint: https://biorxiv.org/cgi/content/short/2023.08.11.552940v1), we demonstrated that axon stiffness affects how oligodendrocyte myelin wrapping responds to promyelinating compounds. We observed that the myelination index differed on artificial axons of superphysiological stiffness compared with physiological stiffness for a given compound. In fact, even the relative ranking among the compounds assayed depended on the relative stiffness of artificial axons. Furthermore, existing technologies such as electrospun fibers^10–14^, enable only indirect measurement of myelin wrapping, as it is challenging to discern whether myelin membrane is merely deposited atop or fully wrapped around these fibers because of their nano-scale diameters, horizontal and poorly aligned architectures.

To address these problems, Espinosa-Hoyos et al. previously developed artificial axons (AAs), a biocompatible platform consisting of 3D-printed hydrogel-based structures made of custom-developed compliant hydrogel (HsP), designed to recapitulate the micrometer-scale diameter and sub-kilopascal mechanical stiffness of biological axons^19,21–23^. Each AA is a free-standing, cylindrical pillar of 20 μm height and ~ 5–8 μm diameter, with stiffness ranging from sub-kilopascal to hundreds of kilopascals. These features mimic biophysical features of biological axons, and are amenable to myelin ensheathment by cultured oligodendrocytes in vitro. Importantly, the combination of the tunable mechanical stiffness of the HsP hydrogel and the flexibility of 3D printing-based fabrication with varied height, diameter, density and spatial arrangements of AAs make this platform highly customizable with capacity to model different disease states.

While the AA fabrication approach reported previously demonstrated proof of concept for compatibility, maturation and visualization of myelin engagement by primary rat OPCs (rOPC), scalability was limited by serial printing of individual coverslips at a rate of approximately two weeks to render each full 96-well plate. This constrained the opportunity to apply this approach for facile comparative analysis of conditions including the multi-well analysis commonly utilized in drug screening. Here we optimize the fabrication of AAs by printing directly into 96-well plates using a near-UV-light based photopolymerization reaction, a fabrication modality that also provides tunability in AA spacing, stiffness, diameter, and geometry. This approach allows for rapid generation of artificial axons with high well-to-well reproducibility, facilitating high throughout phenotypical drug screening.

We harness this AA platform to study a small library of compounds with pro-myelinating activity confirmed in vitro and *in vivo*^9,24–28^, and characterize their dose-dependent effects on rOPC maturation and myelin wrapping. In particular, we developed an image analysis pipeline to quantify the effect of each compound on the propensity and extent of 3D myelin wrapping by oligodendrocytes around the AAs. This analysis pipeline allowed for independent quantification of both the number of axons wrapped and the length of myelin sheaths along each axon. We showed that the effect of these pro-myelinating compounds on myelin wrapping is dose-dependent, and that we can use AA platform to generate characteristic dose–response curves for each compound. Furthermore, we can extract pharmacologically relevant quantities such as the half maximal effective concentration (EC_50_ or potency) and rank the relative efficacy of the compounds. Notably, we found that the ranking of relative efficacies differed substantially between a 3D readout of myelin wrapping, and a 2D readout of myelin basic protein (MBP) area laid by oligodendrocytes grown on the flat surface of tissue culture plates. This result speaks to the importance of studying myelin wrapping in biomimetic formats that can recapitulate the three-dimensional environment that OPCs encounter. In summary, we demonstrate that this AA platform is amenable to drug screening for pro-myelinating compounds, while providing a more biofidelic environment compared to that of existing drug screening technologies.

## Methods

### Ethics statement

This study was carried out in accordance with the guidelines of the National Institutes of Health for animal care and use (Guide for the Care and Use of Laboratory Animals) and the protocol was approved by the Institutional Animal Care and Use Committee at the Massachusetts Institute of Technology (MIT Committee on Animal Care) and at Sanofi. This study is reported in accordance with ARRIVE guidelines (https://arriveguidelines.org).

### Fabrication of artificial axons

We have previously developed a proprietary process to fabricate vertical free-standing artificial axons with diameters of ~ 5–8 μm, heights of ~ 20 μm, and tunable stiffness ranging from sub-kilopascals to ~ 140 kPa, to approximate diameter and stiffness of biological axons^19,21,22^; our prior approaches 3D-printed these polymeric arrays on glass cover slips^19^. In this work, we developed an approach for fast 3D-printing of our custom-developed resin (HsP)^22,23^ directly in the wells of 96-well glass-bottom plates using masked near-UV light and our custom-built 3D printing setup, to enable scalable drug screening. Before cell plating, the AAs were functionalized with poly-D-ornithine (50 μg/ml, 24 h incubation in 37 °C) followed by incubation with laminin (20 μg/ml, 24 h, 4 °C) and stored in 4 °C.

### 3D myelin wrapping assay and dosing with compounds

Rat oligodendrocyte progenitor cells (rOPCs) were isolated from neonatal rat brains (postnatal day 1) using magnetic sorting with beads coated with anti-A2B5 antibodies (Miltenyi, 130–093-392). The isolated cells were expanded in tissue culture flasks for 2–3 days, in the proliferation media (DMEM/F12 (Gibco, 11330-032), Penicilin-Streptomycin (Gibco, 15140-12), B27 (Gibco, 12587-010), PDGF (platelet-derived growth factor, ThermoFisher PHG0035) and FGF (fibroblast growth factor, (ThermoFisher PHG0024) at the concentration of 10 ng/ml each. Expanded rOPCs were plated in 96-well plates containing in each well AAs functionalized with poly-D-ornithine/laminin at a density of 20,000 cells per well of a 96-well plate in 150 μl of the differentiating medium (DMEM/F12, Penicilin-Streptomycin, B27, PDGF and FGF at the reduced concentration of 2 ng/ml each). Only the inner 60 wells were used to avoid the drying effect of the outer-most wells. Plating day was considered day 0 of cell culture. Cells were allowed to attach to AAs for 24 h. Starting with day 1, rOPCs were dosed with compounds every 2–3 days by replacing 50 μl of old media with fresh media containing compound (Monday-Wednesday-Friday schedule), for the total of three repeated doses. Cells were fixed on day 7, followed by immunostaining for myelin basic protein (MBP). For each compound, we applied 9 concentrations obtained by 3 × serial dilutions, starting from the highest concentration of either 10 μM (for benztropine, clemastine, clobetasol, fasudil, ketoconazole, miconazole, quetiapine, and T3), or 1 μM (for tasin-1, tamoxifen, amorolfine, bazedoxifene). As the control condition we used medium containing 0.1% DMSO, which was the solvent vehicle for the compounds. The compounds were provided by Sanofi as 10 mM stock in DMSO. Each condition was repeated in triplicate.

### 2D differentiation assay

In parallel with the 3D myelin wrapping assay, a companion 2D differentiation assay was conducted using the same rOPC cell batch, plate functionalization with poly-D-ornithine/laminin, cell culture and dosing protocol, and assay duration (7 days). For this 2D assay, rOPCs were plated in the 96-well glass bottom plates (60 inner wells) at a density of 10,000 cells per well.

### Enzyme-linked immunosorbent assay (ELISA) analysis of myelin basic protein (MBP)

Standard sandwich ELISA was performed using the following antibodies diluted in PBS (coating antibody) or PBS containing 1% bovine serum albumin (all other antibodies). Coating antibody: monoclonal anti-MBP (1:2500, Millipore Cat# MAB382, RRID:AB_94971); detection antibody: polyclonal anti-MBP (1:2500, Abcam Cat# ab28541, RRID:AB_776581); biotinylated goat anti-rabbit IgG antibody (1:10,000, Vector Laboratories Cat# BA-1000, RRID:AB_2313606), streptavidin-biotinylated HRP complex (1:8000, GE Healthcare, Chicago, USA). Cells harvested at 3- and 6-days post treatment were lysed in triple detergent buffer (50 mM Tris–HCl, pH 8.0, 150 mM sodium chloride, 0.02% sodium azide, 0.1% sodium dodecyl sulfate, 1.0% NP-40, 0.5% sodium deoxycholate, all from Sigma) containing 1X Complete Mini, EDTA-free Protease Inhibitor Cocktail (Roche, Mannheim, Germany). Known concentrations of recombinant bovine MBP (Invitrogen) were used to generate a standard curve. Standards and cell lysates were added to 96-well Maxisorp plates (Nunc, ThermoFisher) pre-coated with coating antibody and incubated overnight at 4 °C. Plates were washed three times with phosphate buffered saline containing 0.5% Tween-20 (Sigma) (PBST) using an automated microplate washer (405 TS, BioTek Instruments Inc. Winooski, USA). Plates were then incubated at room temperature with detection antibody (2 h), biotinylated anti-rabbit IgG (1 h), and streptavidin-biotinylated HRP complex (1 h) with three washes with PBST between each incubation step. To induce colorimetric change, o-phenylenediamine dihydrochloride (OPD) (Sigma) was added to each plate for 30 min and the reaction stopped by addition of 2N sulfuric acid (RICCA). Total MBP concentration was determined by colorimetric change of plates read at 492 nm using the FlexStation^®^ 3 Multi-Mode Microplate Reader (Molecular Devices, San Jose, USA). Total protein concentration in the lysates were determined by Bicinchoninic Acid Assay (BCA) Protein Assay (Pierce, ThermoFisher) according to the manufacturer’s instructions.

### Immunostaining

Cells were fixed with 4% paraformaldehyde (PFA, Electron Microscopy Sciences 15,714-S) in two steps, by first applying 4% PFA in cell media for 15 min, at room temperature (to avoid rapid change from media to PBS), followed by applying 4% PFA in PBS for 15 min. Next, cells were washed 3 times with PBS and permeabilized with 0.2% Triton -X, for 3 min. Cells were then washed 3 times with wash buffer (PBST: PBS/0.01% Tween) and blocked with 5% goat serum in PBST for 1 h. Cells were then incubated with the primary antibody against MBP, the marker of myelin membrane, (BioRad, rat anti-MBP, MCA409, 1:200 dilution) for 24 h, in 4 °C. Next cells were washed 3 times with PBST and incubated with secondary antibody (Alexa-Fluor-647, goat anti-rat, ThermoFisher, A-21247, 1:200 dilution), for 1 h. Cells were then washed 3 times with PBST and incubated with DAPI (ThermoFisher, 62,248, 1:1000 dilution) for 5 min. After washing 3 times with PBST, cells were stored in PBS in in 4 °C.

### Fluorescence imaging

Immunostained samples were imaged either in three channels for AAs samples (rhodamine for AAs, Alexa-Fluor 647 for myelin, and DAPI for nuclei), or in two channels for 2D differentiation samples (Alexa-Fluor 647 for myelin, and DAPI for nuclei) using a confocal microscope (Olympus, FluoView 3000) and 20 × air lens. To image 3D myelin wrapping around axon mimics, for each well of the 96-well plate we collected z-slice images (10 slices, with z-step of 2 μm) in 9 fields of view evenly spaced across the well. Collectively ~ 10,000 AAs were imaged and analyzed per well. For the 2D differentiation samples, single plane images were collected in 9 fields of view per well.

### Quantification of 3D myelin wrapping on AAs

The collected fluorescence z-slice images were processed using Fiji^29^ software and custom-developed protocols to obtain the thresholded binary masks of myelin, axons, and nuclei channels; and the 3D data of myelin wrapping around each axon, including percent of wrapping around axon circumference for each z-slice, and the length of myelin segments along each axon (in z-direction). Using these data, we quantified the number of axons with different percentage of MBP-positive membrane wrapping around axon circumference, for ranges of 0–20%, 20–50%, 50–80%, and 80–100% with at least one z-slice, and a number of axons with 80–100% wrapping and a continuous MBP-positive membrane segment length of at least 6 μm (three z-slices), referred here as “full wrapping”. For each imaged field of view, we quantified “wrapping index”, as the number of fully wrapped axons divided by the number of nuclei. The reported here data are averages over all fields of view.

### Quantification of 2D differentiation

Single-plane images of samples from the 2D differentiation assay were processed using Fiji to obtain thresholded masks of MBP-positive membrane. The area was quantified as a measure of cell differentiation for each field of view. The reported data are averages over all fields of view.

### Compounds potency (EC_50_) and efficacy

The 9-point dose–response data, measured as “wrapping index” for 3D myelin wrapping assay, or as MBP area for 2D differentiation assay, were fitted to sigmoidal curve model using Origin Pro (OriginLab Corporation) data analysis software. The reported values of compound’s potency—EC_50_ or the effective compound’s concentration to induce 50% of maximum effect—were obtained from the fitted curves. Efficacy (the maximum magnitude of wrapping index) was reported directly from the obtained data points, rather than from a fit to all data points in the dose response. Relative efficacy for each compound was defined as maximum wrapping index magnitude for that compound, divided by the maximum wrapping index in response to T3.

### Statistical analysis

The 3D myelin wrapping and 2D differentiation assays to obtain dose responses to compounds were performed in triplicate (3 wells). For each well we analyzed images from 9 fields of view per well, with ~ 10,000 AAs analyzed for the 3D myelin wrapping experiment and MBP-positive area analyzed for the 2D differentiation experiment. The reported data are averages over all fields of view. One-way ANOVA was used to determine statistical significance of differences between each pair of compared conditions.

## Results and discussion

### Biomimetic AA assay enables quantification of 3D myelin wrapping in neuron-like environment

**Figure 1:**
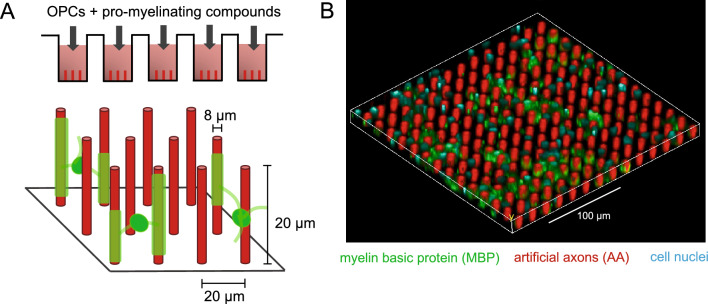
AA myelin wrapping assay. (**A**) Schematic of oligodendrocytes wrapping myelin membrane around AAs, and (**B**) confocal micrograph showing AAs ensheathed by myelin basic protein (MBP).

**Figure 2:**
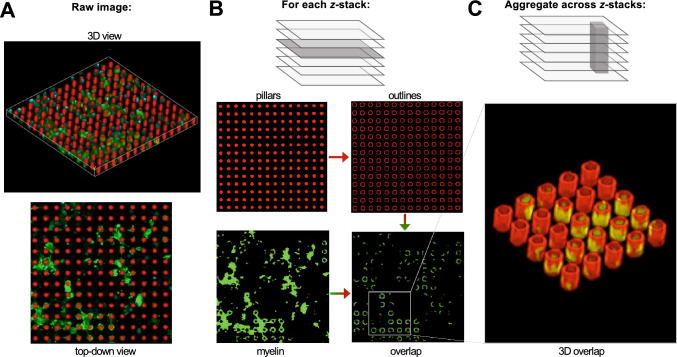
(**A**) Confocal micrograph of myelin basic protein (green) wrapped around artificial axons (red) (**B**) Top-down binary mask generated for each *z-*slices, used to generate an overlap mask between the pillar and myelin channels (**C**) Aggregated overlap masks across all *z*-slices to quantify wrapping across the three-dimensional extent of each axon.

We previously fabricated AAs via projection microstereolithography^19,21,22,30^, a 3D printing technique that uses patterned UV light to photopolymerize micron-scale structures. We also previously developed a custom poly(HDDA-*co*-starPEG) resin^22,23^ that is compatible with this fabrication modality; the resin is liquid in its unpolymerized form, but exposure to UV light induces polymerization resulting in a columnar geometry^31^. Here we build on this prior work by developing a new 3D printing system and workflow for fabricating AAs directly inside 96-well plates, shown in Fig. 1A,B. To fabricate the AAs, UV light is projected through a photomask, causing the resin to polymerize into vertical pillars within an array that spans the entire surface area of each well within a 96-well plate upon UV exposure (Fig. 1A). In our myelin wrapping assay, each AA is a free-standing, ~ 20 μm tall cylindrical pillar with uniform diameters of ~ 8 μm and stiffness ~ 140 kPa (Young’s modulus) that is amenable to myelin ensheathment by cultured oligodendrocytes in vitro (Fig. 1A). Each AA-containing well is seeded with primary rat oligodendrocyte progenitor cells in cell culture media. The cells are treated every 2–3 days with total of three doses of a pro-myelinating compound. After seven days of rOPC culture the cells were fixed and stained against myelin basic protein (MBP), and quantified for myelin wrapping around AAs using confocal microscopy. Figure 1B shows an oblique view of MBP-containing myelin (green) wrapped around and deposited among rhodamine-stained AAs (red). The uniquely designed vertical axon geometry is amenable to automated quantification and enables a direct readout of the extent of myelin wrapping around the AA’s circumference, and the myelin sheath length (Fig. 2).

### Quantification of the myelin wrapping index

To quantify the extent of myelin ensheathment from confocal micrographs (Fig. 2A), we generated a binary mask at each *z*-slice for the rhodamine-stained AA and myelin channels (observed by immunofluorescence staining for myelin basic protein, MBP) (Fig. 2B). For each AA mask, we traced a 1 px-thick outline around each AA. We then generated an ‘overlap mask’ by comparing the AA outline tracing with the corresponding pixels in the myelin mask. Pixels in the outline that were also myelin-positive were assigned a value of 255 in the overlap mask, and outline pixels with no corresponding myelin were assigned a value of 0 in the overlap mask. Therefore, the overlap mask is a binary image that captures the fractional circumference of each AA with myelin membrane around it. Finally, we compared the overlap masks across all *z*-slices, thus aggregating the myelin coverage of each discrete ensheathed segment along the length of each AA (Fig. 2C). We defined an AA as being ‘fully wrapped’ if there was a contiguous > 6 μm ensheathed segment.

length in which the AA was > 80% wrapped in all of those *z*-slices. We note that it is not yet confirmed whether the observed wrapped MBP-positive membrane exhibits the structural features of compact myelin. Scanning electron microscope imaging remains underway to visualize such nano-scale features in this hybrid cell-synthetic polymer assay.

This image analysis pipeline uniquely enabled us to obtain 3D readouts of myelin wrapping. We defined a ‘wrapping index’ parameter, which was the number of AAs exhibiting at least one fully wrapped segment, divided by the total number of cells in the field of view. We determined the total oligodendrocyte number by counting the number of DAPI + nuclei observed. Normalizing by cell number in this way allowed us to account for subtle variations in cell density between different regions of the well. Whereas wrapping index is a measure of the *number* of AAs, we also quantified the *length* of myelin sheaths on those wrapped AAs (see Fig. 6).

### Phenotypic AA 3D myelin wrapping assay reveals dose-dependent response to pro-myelinating compounds

**Figure 3:**
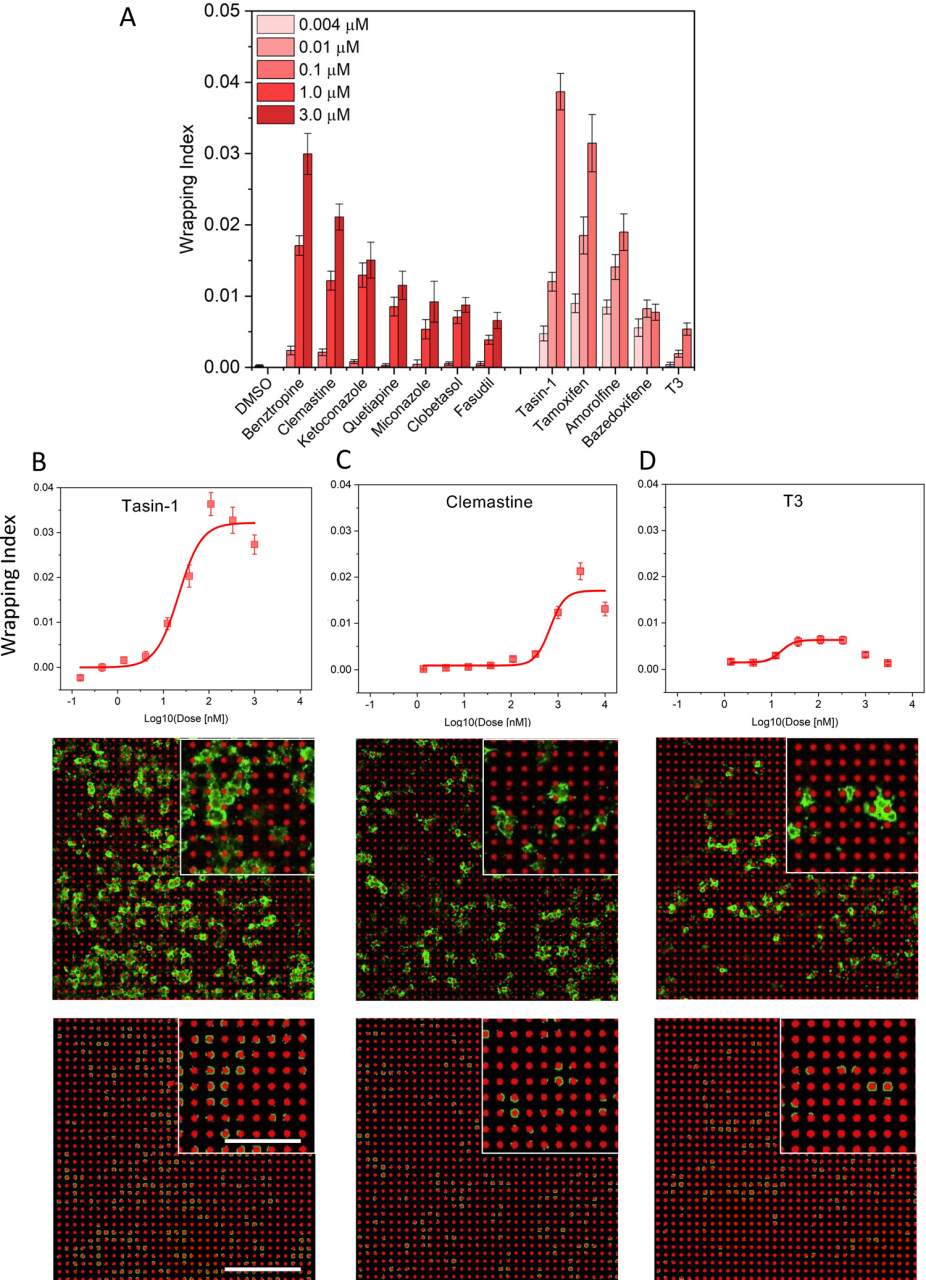
Dose-dependent myelin wrapping of AAs in response to compounds. (**A**) 3-point series of concentration (taken from 9-point dose response data) illustrate compounds dose-dependent effect on myelin wrapping. (**B**–**D**) Top panel: examples of sigmoidal dose–response curves fitted to the wrapping index data for the 9-point concentration series, for, tasin-1, clemastine and T3. The curves for all the tested compounds are shown in Supplementary Fig. S1; The sigmoidal fit excludes concentrations above which the drug induced cytotoxicity. Reported are average values over all fields of view (3 replicates with combined 27 fields of view). Error bars are standard error of the mean (SEM); Middle panel: examples of MBP staining of myelin wrapping around artificial axons (shown is one field of view at a selected z-plane) corresponding the maximum efficacy dose for tasin-1 (111 nM), clemastine (3 µM) and T3 (111 nM); Bottom panel: processed images from middle panel showing wrapped myelin membrane around artificial axons. Scale bars: main image: 200 μm, insert: 100 μm.

Current in vitro methods to screen for compounds with pro-myelinating potential rely mostly on 2-dimentional differentiation assays that quantify either the area of myelin membrane laid by oligodendrocytes on the flat surface of a tissue culture plastic or the amount of expressed myelin proteins such as MBP. More recently, “quasi” 3-dimensional assays using layers of electrospun fibers made of stiff polymers and deposited on a 2D substrate surface have also been used, providing readouts of the alignment of oligodendrocyte processes with the fibers. These assays do not directly measure the compound’s potential to promote actual myelin wrapping, and they exhibit material stiffness several orders of magnitude higher than that of neuronal axons. In contrast, AAs enable a direct readout of 3D myelin wrapping by oligodendrocytes in a closer approximation of biological axon geometry and stiffness. We tested a small library of compounds with demonstrated potency to increase expression of MBP by rat oligodendrocyte precursor cells, to assess whether the platform could resolve dose-dependent propensity for 3D wrapping of AAs. The EC_50_ values of these compounds were established to be within the nanomolar to micromolar range when MBP expression was quantified by ELISA (Table 1). We considered two subsets of compounds: group A comprising compounds with measured MBP-ELISA EC_50_ values within the range from 100 nM to 1 μM, and group B comprising compounds with measured MBP-ELISA EC_50_ below 100 nM. For each compound, we computed the wrapping index (see “Methods”), to generate a nine-point dose–response curve (Fig. 3), which was fitted with a sigmoidal curve, allowing for calculation of the EC_50_ and efficacy (the maximum effect) of the compounds’ promyelinating potential in vitro (Fig. 3B–D, Supplementary Fig. S1, Table 1). Our AA assay demonstrated well-defined dose-dependent myelin wrapping for almost all tested compounds. Only one compound (fasudil) did not exhibit a response plateau/maximum at the highest tested concentration of 10 μM (Fig. S1). To our knowledge this is the first in vitro platform that enables measuring the dose-dependence curves for myelin wrapping in response to compounds. In the present 3D myelin wrapping assay, all tested compounds demonstrated higher efficacy than the vehicle DMSO and T3, the extensively validated promyelinating thyroid hormone (Table 1). The highest promyelinating efficacy we measured for tasin-1, tamoxifen, and benztropine. The EC_50_ values quantified by our assay’s wrapping index were within an order of magnitude of the concentrations obtained from MBP-ELISA. We refrain from any speculative comparison of the relative EC_50_ between these assay types, as the reported here ELISA experiments were conducted separately from the present AA assays, using different batches of rOPCs.

**Table 1:** Relative efficacy (with respect to T3 response), rank, and EC_50_ (nM) and for compounds obtained for 3D myelin wrapping assay using AAs, 2D differentiation assay, and ELISA.

Compound	Relative efficacy (relative to T3 response) and rank				EC_50_, nM		
	3D AAs		2D glass		3D AAs	2D glass	ELISA* (MBP expression)
	Efficacy	Rank	Efficacy	Rank			
Tasin-1 (B)	7.8 (0.5)	1	2.0 (0.2)	2	22	13	17
Tamoxifen (B)	6.4 (0.8)	2	2.0 (0.2)	2	8	2	88
Benztropine (A)	6.1 (0.6)	2	2.1 (0.1)	2	581	375	127
Clemastine (A)	4.3 (0.4)	3	1.4 (0.1)	Below T3 control	678	121	120
Amorolfine (B)	4.1 (0.4)	3	2.4 (0.2)	1	5	10	16
Ketoconazole (A)	3.0 (0.5)	4	1.6 (1.1)	3	507	315	470
Fasudil (A)	2.5 (0.3)	5	1.0 (0.1)	Below T3 control	–	339	260
Clobetasol (A)	2.4 (0.5)	5	1.6 (0.2)	3	1040	546	455
Quetiapine (A)	2.3 (0.4)	5	1.9 (0.2)	2	635	207	184
Miconazole (A)	1.9 (0.6)	6	1.5 (0.1)	3	623	397	302
Bazedoxifene (B)	1.8 (0.2)	6	0.6 (0.1)	Below T3 control	3	0.1	16
T3 (B)	1.0 (0.2)	7	1.0 (0.1)	5	16	7	96

Uncertainty in relative efficacy is expressed parenthetically as standard error of the mean (SEM).

Group (A)—compounds in this group had measured MBP-ELISA EC_50_ values within the range from 100 nM to 1 μM.

Group (B)—compounds in this group had measured MBP-ELISA EC_50_ below 100 nM.

*ELISA experiments were performed with a different batch of rOPCs than used in the 3D AA and 2D differentiation experiments.

### The 2D oligodendrocyte differentiation assay is not a good predictor of compounds relative 3D pro-myelination potential

**Figure 4:**
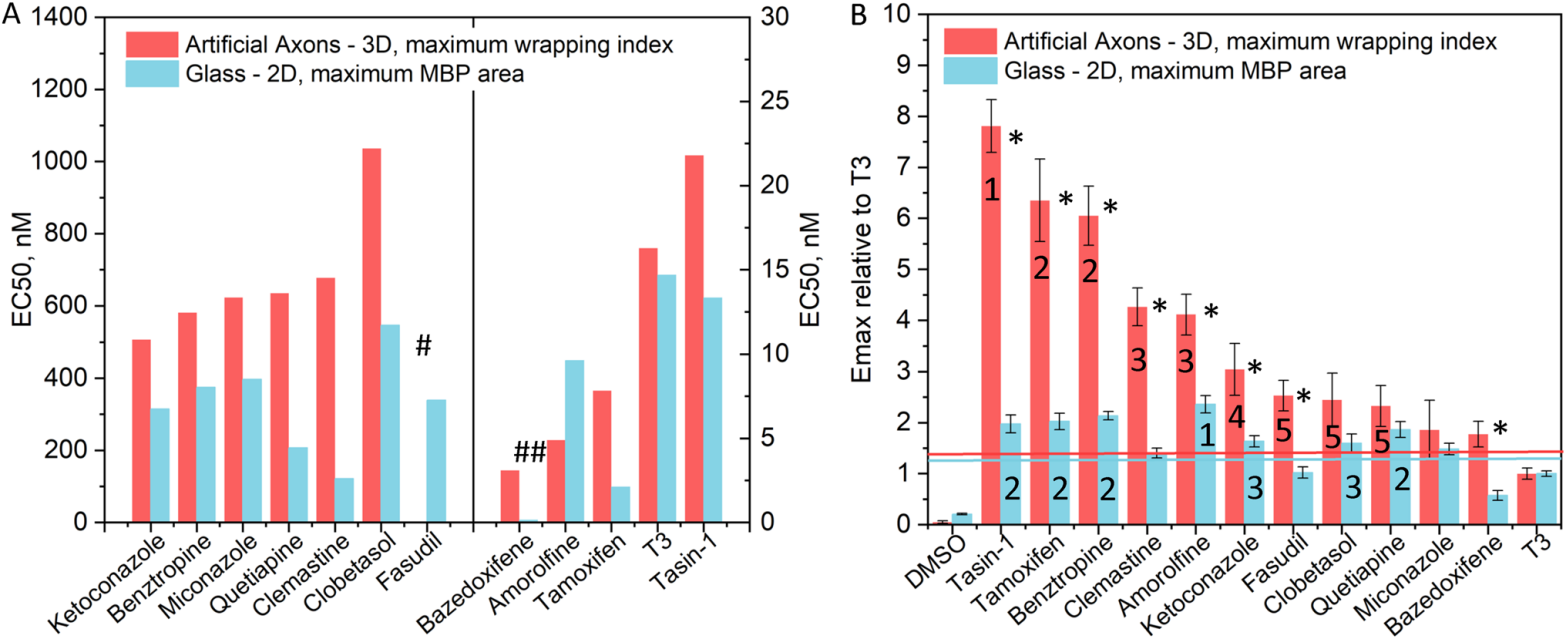
(**A**) The EC_50_ values (left scale for the compound subset left from the middle line and right scale for the compound subset right from the middle line) and (**B**) the efficacy values relative to T3, obtained from 9-point dose–response data for wrapping index in 3D myelin wrapping assay (red columns) and for MBP area in 2D differentiation assay (blue columns). The compounds are organized according to the values for 3D wrapping assay, for easier visualization of the different outcomes from the myelin wrapping and differentiation assays. Reported are average values over all fields of view (3 replicates with combined 27 fields of view). ^#^EC_50_ for fasudil in 3D AA assay was not determined due to the lack of plateau of dose response curve at high dose range; ^##^EC_50_ for bazedoxifene in 2D differentiation assay: 0.1 nM. In (**B**) error bars are standard error of the mean (SEM), numbers denote rank, horizontal lines denote 2 × standard deviation for T3 in the respective 3D and 2D assays; In (**A**) EC_50_ values are obtained from one experimental screen with 3 replicates resulting in one dose–response curve, therefore no SEM is available. *p-value < 0.05 between 3 and 2D conditions for each compound.

Traditionally, the capacity of compounds to stimulate myelin wrapping in vitro is inferred from 2D differentiation assays. Such 2D assays quantify the relative amount of MBP production, the marker of OPC maturation and differentiation into myelinating oligodendrocytes, rather than the active process of 3D ensheathment by the MBP-containing myelin. Although differentiation is a required step in oligodendrocyte biology to reach a mature stage of the myelinating oligodendrocyte, differentiation and myelin wrapping are distinct processes. We speculated that the compounds’ relative ranking could differ for pro-differentiation vs. pro-myelination capacities. We thus conducted a conventional 2D differentiation assay in parallel with the AA assay, using the same preparation of rOPCs. We performed the differentiation assay using glass-bottom 96-well plates, to match the commonly used conditions for this kind of assay. For most of the compounds, the EC_50_ determined by the wrapping index was noticeably higher than that determined by the 2D MBP area, indicating a “lag” of actual myelin wrapping with respect to the differentiation and production of MBP-positive membrane (Fig. 4A). In other words, for these compounds a higher dose is needed to reach the half-effect on myelin wrapping than on differentiation. The only compound in our assay with the reverse order of EC_50_ was amorolfine. The rank order of compounds based on their EC_50_ was also different for the myelin wrapping assay compared to differentiation (Fig. 4A). The comparison of the relative efficacy (i.e., maximum wrapping index magnitude for each compound, divided by the maximum wrapping index in response to T3) also shows differences between the 3D and 2D assays (Fig. 4B). First, the ranking of compounds is different, with the top three compounds in the 3D wrapping assay being tasin-1, tamoxifen and benztropine; in the 2D assay, the top compound was amorolfine, followed by several compounds (quetiapine, benztropine, tamoxifen and tasin-1), for which the response could not be distinguished statistically (Fig. 4B and Table 1). Second, in the 2D assay, the compounds bazedoxifene, fasudil and clemastine fell below the control threshold and would thus be missed, whereas clemastine and fasudil showed strong signal in the wrapping assay. Finally, the wrapping assay was able to clearly differentiate among most compounds, whereas in the 2D assay many compounds exhibited indistinguishable differences and performance. For example, tasin-1, tamoxifen, benztropine, and quetiapine induced indistinguishable performance in the differentiation assay, but all of those compounds exhibited distinct pro-wrapping properties in the 3D wrapping assay. These differences would not have been predicted from the 2D differentiation assay.

Overall, the 3D wrapping assay reveals much larger relative differences between the compounds than the differentiation assay, in addition to a different relative ordering of the compounds’ efficacy. In conclusion, the interpretation of compounds’ relative ability to promote myelin wrapping inferred from differentiation assay is different from our AA assay, which directly quantifies 3D myelin wrapping around axon mimics. This suggests that the 2D differentiation assays that quantify myelin membrane area are not good predictors of compounds relative pro-myelination potential.

### Changes of myelin wrapping extent with increasing compound dose differ among compounds

**Figure 5:**
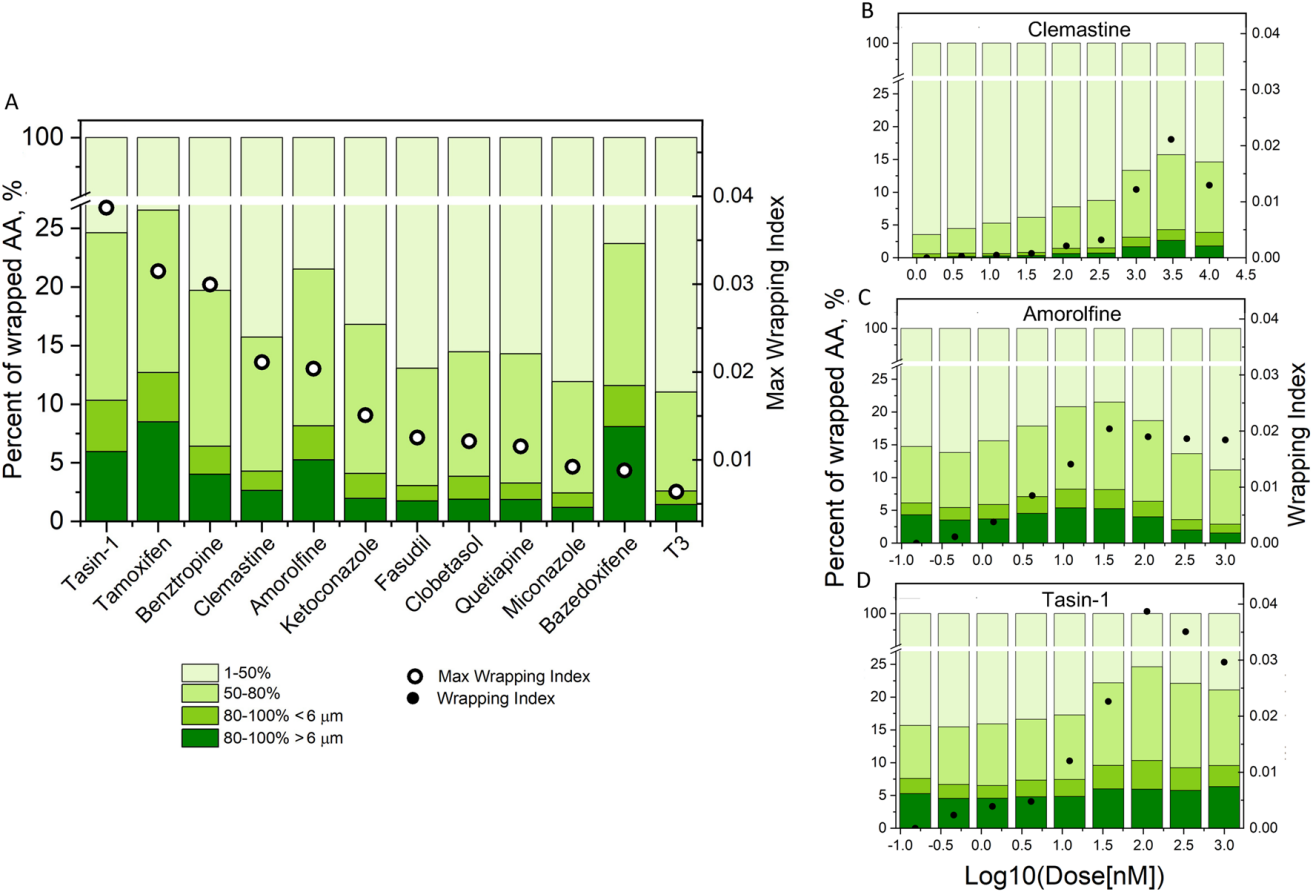
Changes of myelin wrapping extent with compounds concentration. (**A**) Percentage of AAs with increasing extent of wrapping (< 50%, 50–80%, 80–100% and 80–100% with > 6 µm myelin segment; left axis scale) for each compound at its maximum effect (max wrapping index) (green columns). Mapped on it is maximum wrapping index for each compound (open circles, right axis scale). (**B**–**D**) Examples of wrapping progression expressed as the same percentages as in (**A**) for increasing compound concentration, for clemastine, amorolfine, and tasin-1, to illustrate different progression profiles (green columns, left axis scale). Mapped on it is wrapping index at each concentration (black dots, right axis scale). See Supplementary Fig. S2 for all tested compounds.

For each compound (at the dose corresponding to its highest wrapping index) we considered all the AAs that exhibited any MBP-positive membrane on their surface (0–100% of the AA circumference). Within this set we quantified the percent of AAs at different stages of wrapping, from the least (< 50% of AA circumference) to the most engaged or fully wrapped (> 80% of AA circumference with the MBP-positive segment length > 6 μm) (Fig. 5A). Note that the darkest green region for each response bar represents the percentage of fully wrapped AAs within the considered AAs subset. This morphological analysis revealed further differences among the compounds, showing the highest fraction of fully wrapped AAs for tamoxifen, bazedoxifene, tasin-1, and amorolfine. We also analyzed how the wrapping progressed with increasing dosage (Fig. 5B–D, and Supplementary Fig. S2). The examples in Fig. 5B–D show three different types of dose response. Clemastine (Fig. 5A) induced a gradual increase in the percentage of fully wrapped AAs with increasing drug dosage; amorolfine induced this maximum in the middle of this dose range; and tasin-1 induced a dosage-independent percentage of fully wrapped AAs. Similar plots for all the compounds are shown in Supplementary Fig. S2.

### 3D analysis of myelin sheath length

**Figure 6:**
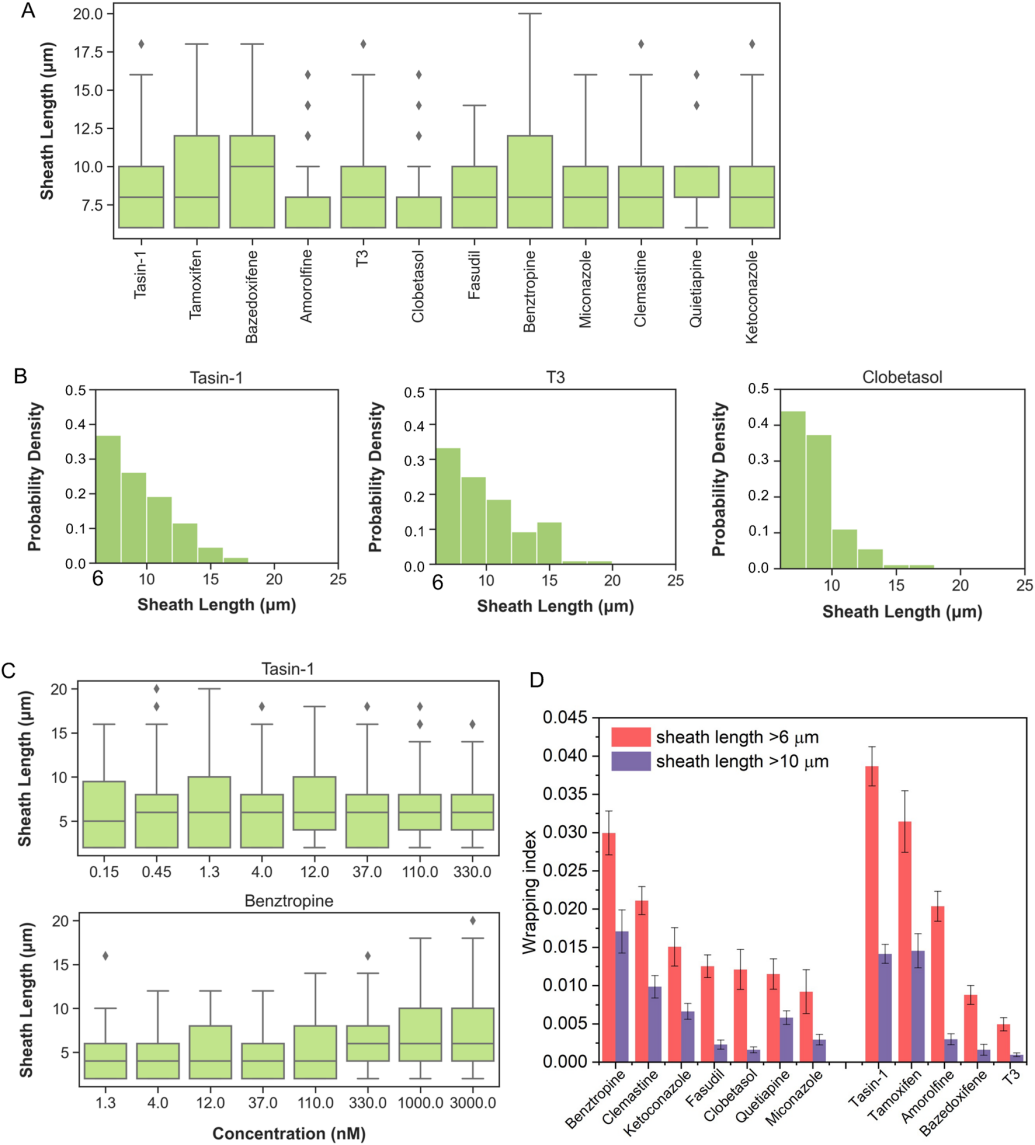
(**A**) Box plots of the length distributions of myelin sheaths with > 80% of wrapped circumference at the concentration corresponding to the maximum wrapping index for each compound. (**B**) Normalized histograms showing differences in the length distribution of myelin sheaths > 6 µm, for tasin-1, T3, and clobetasol. Histograms were generated for concentrations corresponding to the maximum wrapping index (111 nM for tasin-1 and T3, and 3 μM for clobetasol). (**C**) Box plots showing the length distribution of myelin sheaths with > 80% of wrapped circumference for tasin-1 and benztropine at different drug concentrations. Box represents the interquartile range of 25 (Q1)-75 (Q3) percentile (IQR), whiskers represent data set minimum (Q1-1.5*IQR) and maximum (Q3 + 1.5*IQR), diamonds represent individual outliers, middle line represents data set median. (**D**) Comparison of maximum wrapping index for myelin sheath length > 6 µm (red) and > 10 µm (violet). Error bars are standard error of the mean (SEM) (for 3 replicates with combined n = 27 fields of view).

Another unique advantage of AAs is the capacity to quantify the segment length of discrete myelin sheaths along each AA. In Fig. 6A, we compared the sheath length distributions and medians for all compounds, at the concentration corresponding to the maximum of drug effect (maximum wrapping index). The median sheath length was similar among all the compounds (~ 8 μm), except bazedoxifene, which induced on average longer sheaths (~ 10 μm), despite having relatively low efficacy as measured by the maximum wrapping index, which quantifies the number of fully wrapped axons.

The ability to induce longer sheaths was also noted for tamoxifen and benztropine, which is indicated by the distributions reaching larger length values (Fig. 6A). Further histogram analysis of the sheath lengths > 6 μm revealed more detailed differences among these compounds (Fig. 6B). Figure 6B shows examples of the length distributions for tasin-1 and T3 at 111 nM concentration, and for clobetasol at 3 μM, the doses corresponding to the maximum wrapping index. Similar plots for all the compounds are shown in Supplementary Fig. S3. Even though tasin-1 and T3 exhibited substantially different efficacies as measured by maximum wrapping index (Fig. 4), they both showed rather similar distribution of sheath lengths. In contrast, clobetasol induced less sheaths longer than 10 μm, compared to tasin-1 and T3. One might expect a priori that the sheath length would increase with increasing drug concentration. However, unlike wrapping index that quantified the cell-normalized *number* of fully wrapped AAs, the average sheath *length* of those wrapped AAs did not exhibit any dose-dependence for most of compounds considered, similar to shown here example of tasin-1 (Fig. 6C, Supplementary Fig. S3). An exception was benztropine, for which we observed induction of longer median and maximum sheath lengths with increasing concentration (Fig. 6C). This finding suggests that the primary effect of most of the tested here compounds is to influence the *number* of fully wrapped AAs rather than the MBP-positive sheath *lengths* along those AAs. The quality of drug response could also be reasonably considered to include the sheath length, with the tacit expectation that longer spans of myelin along the neuronal axon (up to a limit of internodal length) is a superior response. To consider whether quantitation of sheath length would affect relative efficacy among compounds, we varied the threshold of fully wrapped segment length. Figure 6D illustrates this point for a wrapping index that uses a 10 μm segment length threshold (violet bars) instead of the 6 μm threshold adopted throughout the prior analysis. In general, drugs that had a higher maximum wrapping index for 6 μm sheath lengths (red bars) also showed higher numbers of fully wrapped AAs with > 10 μm sheaths. However, comparing the two metrics side-by-side in this way can enable identification of disparate responses among drugs, such as to amorolfine, clobetasol and fasudil. Even though these three compounds induced high numbers of AAs wrapped with > 6 μm sheaths, none of these three compounds scored well if the threshold for full wrapping was 10 μm sheath length.

We note that because of the short assay duration (7 days) and relatively short axon lengths (20 μm) in this assay designed to maximize speed while achieving quantitative differentiation among the drugs’ pro-myelinating activity, matured myelination was not observed. For example, average sheath lengths in our assay were shorter than the physiologically observed average sheath lengths in rodent CNS (~ 30 μm)^10,32^, although we note that many CNS axons have sheath lengths much shorter than 30 μm. This is not a limitation of the platform but rather a choice of this particular drug screening assay design. In fact, in our previous work (Doctoral Thesis, D. Espinosa-Hoyos, MIT, 2020) using a different artificial axon array format, we demonstrated that the sheath length distribution reproduced the distribution observed in vivo*.*

Taken together, this analysis pipeline allows us to decouple the *number* of AAs wrapped from the *length* of myelin sheaths on those wrapped AAs. By providing different measures to evaluate myelin wrapping extent, such as the number of wrapped AAs and sheath length, this platform exposes differences in pro-myelinating properties among compounds that are not accessible in other in vitro assays.

## Summary

Here, we developed and validated the phenotypic 3D myelin wrapping assay using the artificial axons platform by evaluating its ability to quantify the pro-myelination potential of a small library of known pro-myelinating compounds. This platform consists of 3D-printed hydrogel-based axon mimics that recapitulate the micrometer-scale diameter and kilopascal-scale mechanical stiffness of biological axons, providing physiologically relevant environment for myelinating oligodendrocytes, unmatched by other existing in vitro drug screening myelination platforms such as glass cones or electrospun fibers, which have mechanical stiffness at least five orders of magnitude higher than that of biological axons. These artificial axons can be readily wrapped with MBP-positive myelin membrane by cultured oligodendrocytes in vitro. We showed that the effect of these pro-myelinating compounds on myelin wrapping is dose-dependent, and that we can use AAs to generate characteristic dose–response curves for each compound to determine in vitro EC_50_ and efficacy for myelin wrapping, from sub-nanomolar to micromolar concentrations.

Notably, we found that the ranking of efficacy and myelination potency (EC_50_) among compounds differed significantly between this AA myelin wrapping assay and the corresponding conventional oligodendrocyte differentiation assay (planar deposition of the MBP-positive membrane) (Fig. 4A,B). We showed that these differences could lead to missing identification of some compounds that can promote myelin wrapping if they were screened only in a 2D differentiation assay. Moreover, the dynamic range or relative difference among compounds as quantified by efficacy using the AA myelin wrapping assay was significantly greater than that of the planar differentiation assay (Fig. 4B). This heightened differential response in the functional result-of-interest (axon engagement and wrapping by the oligodendrocyte) increased the signal-to-noise ratio, potentially helping to discriminate among the compounds, which were indistinguishable in the differentiation assay.

Further, this three-dimensional analysis of axon wrapping allows for quantification of myelin sheath length, which is related to the physiological internode length. Interestingly, we observed no significant correlation of the sheath length with compound concentration for most of tested here compounds (with exception of benztropine) (Fig. 6B and Supplementary Fig. S3). However, we did observe differences among sheath length distributions (Fig. 6C and Supplementary Fig. S3) and the compounds’ potential to induce sheath lengths > 6 and > 10 μm (Fig. 6D). Specifically, we observed that three compounds (tasin-1, tamoxifen and benztropine) showed a greater propensity to induce more axons wrapped with longer myelin sheath lengths. Previous in vivo and ex vivo quantification of myelin sheath length distributions in murine cell co-culture and brain tissue slice analysis indicates a mean sheath length greater than 10 μm^10,32^. Thus, these unique readouts of the AA wrapping assay such as sheath length has the potential to improve selection of compounds with the ability to induce more physiological wrapping.

Although in this study, which was focused on demonstrating the platform’s potential for promyelinating drug discovery and evaluation, we used one specific configuration of artificial axons (dimeter of ~ 8 μm, height of ~ 20 μm, axon center-to-center spacing of 20 μm, and stiffness ~ 140 kPa), the unprecedented potential of this platform is in the ability to vary each of these parameters independently to mimic specific physiological or disease conditions. We explored this potential in the separate study, considering how variation of these parameters influences myelin wrapping and responses to compounds (preprint: https://biorxiv.org/cgi/content/short/2023.08.11.552940v1).

In conclusion, we demonstrated that the artificial axons platform is amenable to high-throughput drug screening for pro-myelinating compounds, enabling direct quantification of 3D myelin wrapping extent and sheath length within a biofidelic environment.

### Supplementary Information

Supplementary Figures.

## Data Availability

The datasets used and/or analysed during the current study available from the corresponding author on reasonable request.
