# Supplementary material for: Artificial axons as a biomimetic 3D myelination platform for the discovery and validation of promyelinating compounds
Source: Sci Rep. 2023 Nov 9;13:19529. doi: 10.1038/s41598-023-44675-6 (PMC10636046; doi:10.1038/s41598-023-44675-6)

SUPPLEMENTARY MATERIALS

Artificial axons as a biomimetic 3D myelination platform for the discovery and validation of promyelinating compounds

Anna Jagielska[1,*], Kristin Radzwill[2], Daniela Espinosa-Hoyos[3], Mingyu Yang[4], Kavin Kowsari[5], Jonathan E. Farley[2], Stefanie Giera[2], Ann Byrne[2], Guoqing Sheng[2], Nicholas X. Fang[5], James C. Dodge[2], Carlos E. Pedraza[2], and Krystyn J. Van Vliet[1, 6,*].

Authors Affiliations:

1. Department of Materials Science and Engineering, Massachusetts Institute of Technology, Cambridge, MA; 2. Sanofi, Cambridge, MA; 3. Department of Chemical Engineering, Massachusetts Institute of Technology, Cambridge, MA; 4. Harvard-MIT Health Sciences and Technology, Massachusetts Institute of Technology, Cambridge, MA; 5. Department of Mechanical Engineering, Massachusetts Institute of Technology, Cambridge, MA; 6. Department of Biological Engineering, Massachusetts Institute of Technology, Cambridge, MA.

* **Corresponding Authors:** Correspondence and requests for materials should be addressed to A. Jagielska (anna.jagielska@gmail.com) and K. J. Van Vliet (krystyn.vv@cornell.edu).

Figure S1. Dose response curves for all compounds. Sigmoidal dose-response curves fitted to the wrapping index data for the 9-point concentration series. The fit excludes concentrations above which the drug induced cytotoxicity. Reported are average values over all fields of view (3 replicates with combined 27 fields of view). Error bars are SEM.

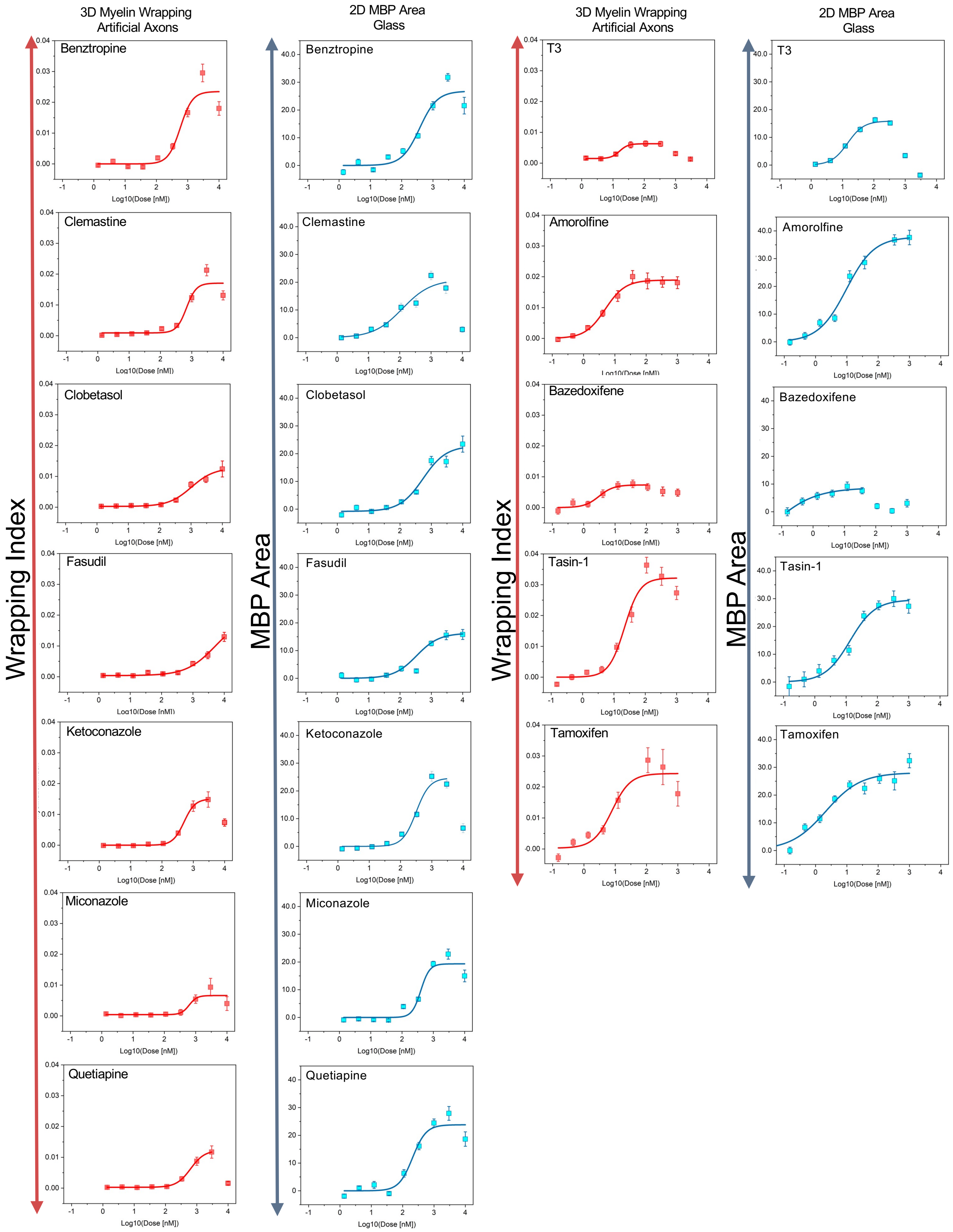

Figure S2. Wrapping changes with increasing concentration for all compounds. Percentage of AAs with increasing extent of wrapping (<50%, 50-80%, 80-100% and 80-100% with >6 μm myelin segment; left scale) for increasing compound concentration. Mapped on it is wrapping index at each concentration (black dots; right scale).

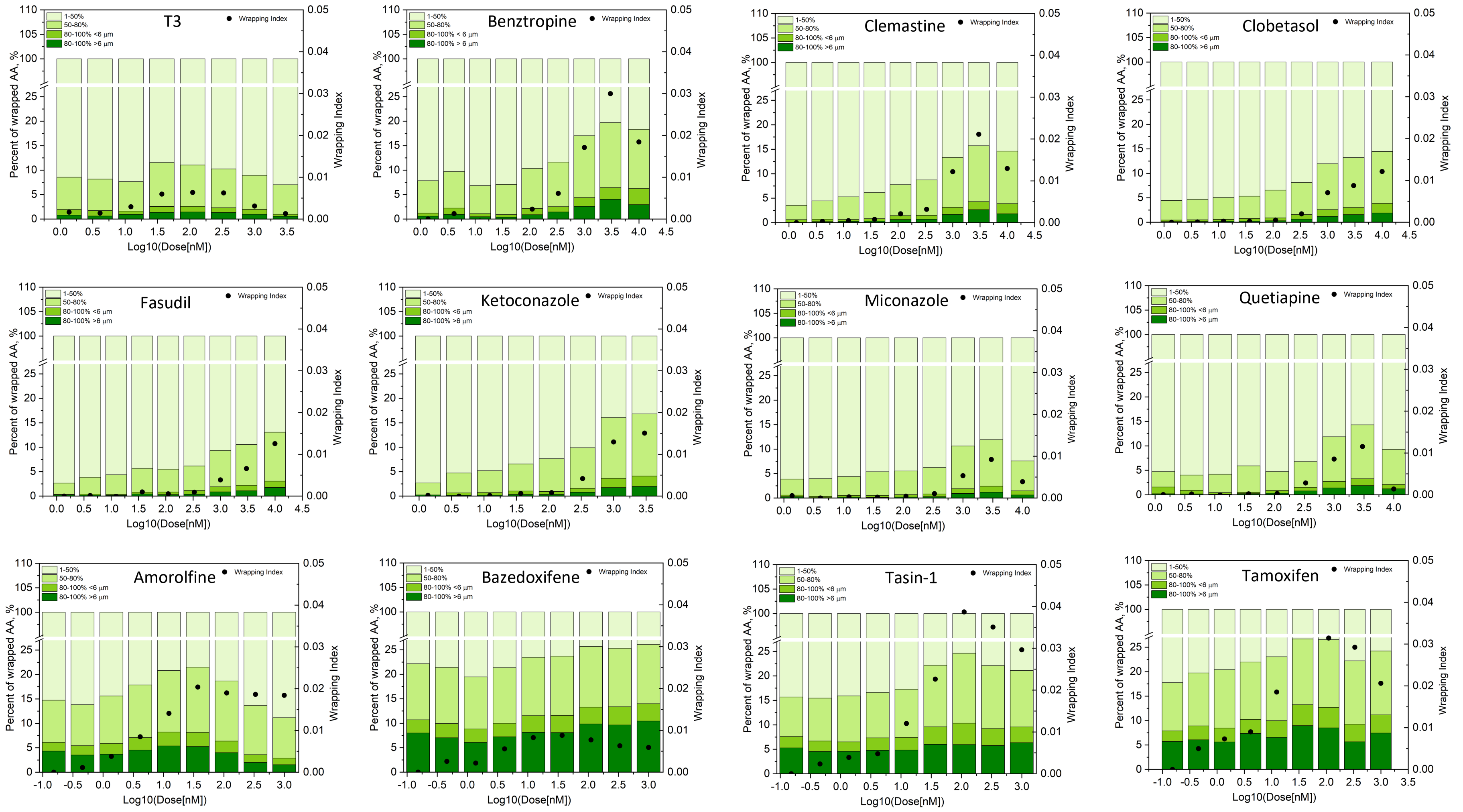

Figure S3. Length analysis for all compounds. (A) Normalized histograms showing the length distribution of myelin sheaths >6 μm for all compounds. Histograms were generated for concentrations corresponding to the maximum wrapping index for each compound. (B) Box plots showing the length distribution of myelin sheaths different drug concentrations. Box represents the interquartile range of 25 (Q1)-75 (Q3) percentile (IQR), whiskers represent data set minimum (Q1-1.5*IQR) and maximum (Q3+1.5*IQR), diamonds represent individual outliers, middle line represents data set median.

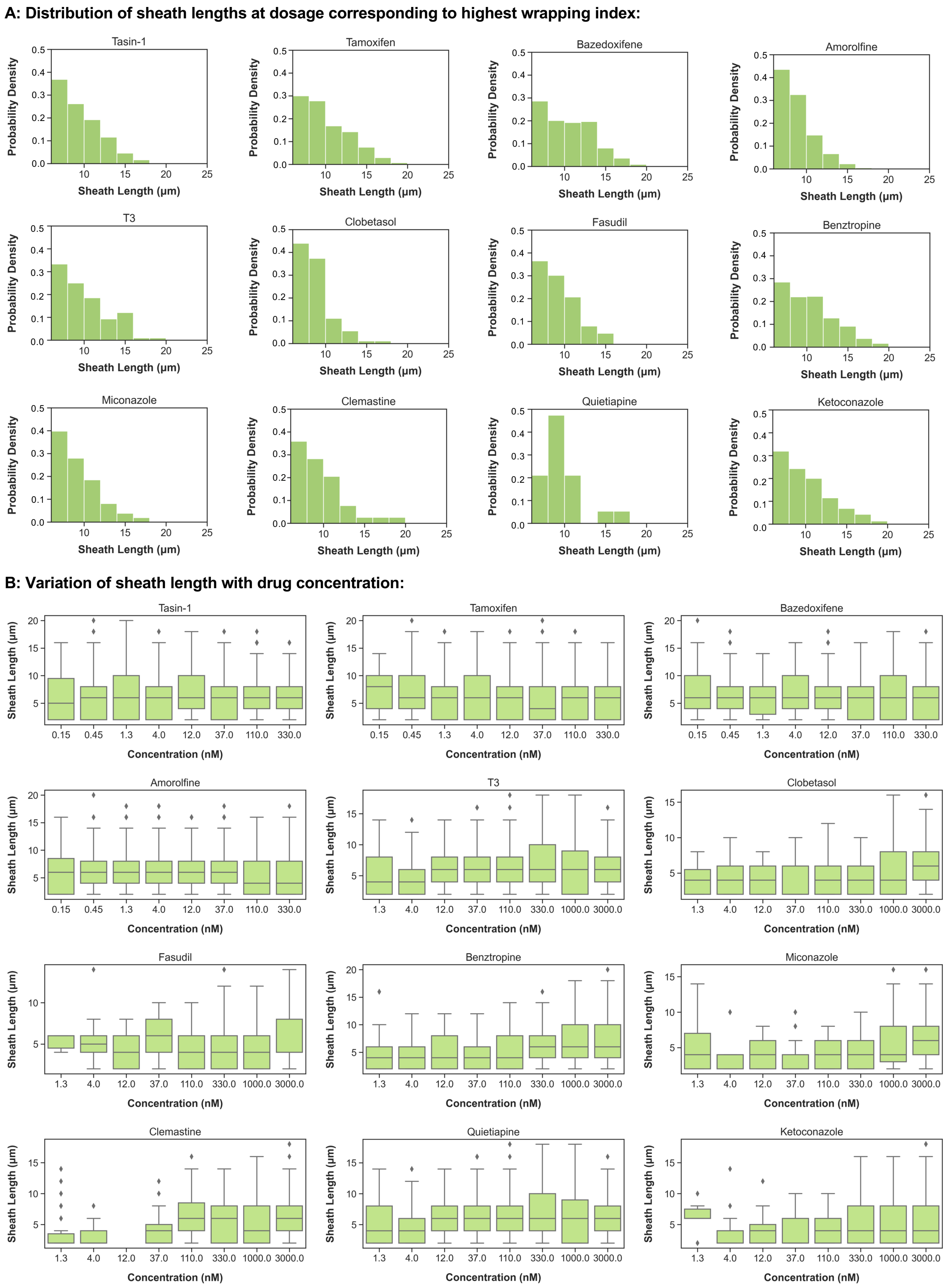

Supplement: Supplementary file 1 — Supplementary Figures. [file 41598_2023_44675_MOESM1_ESM.pdf]
